# Coarse-Fine-Stitched: A Robust Maritime Horizon Line Detection Method for Unmanned Surface Vehicle Applications

**DOI:** 10.3390/s18092825

**Published:** 2018-08-27

**Authors:** Yuan Sun, Li Fu

**Affiliations:** 1 School of Electronics Engineering, Beijing University of Posts and Telecommunications, Beijing 100876, China; 2 Beijing Institute of Aerospace Control Devices, Beijing 100094, China; li.fu@buaa.edu.cn

**Keywords:** horizon line detection, unmanned surface vehicle, maritime application, robust detection, vision sensor

## Abstract

The horizon line has numerous applications for an unmanned surface vehicles (USV), such as autonomous navigation, attitude estimation, obstacle detection and target tracking. However, maritime horizon line detection is quite a challenging problem. The pixel points of the horizon line features are far fewer than the pixel points of the entire image, on the one hand. Conversely, the detection results might be impacted negatively by the complex maritime environment, waves, light changing, and partial occlusions due to maritime vessels or islands, for example. To solve these problems, a robust horizon line detection method named coarse-fine-stitched (CFS) is proposed in this paper. First, in the coarse step of CFS, a line segment detection approach using gradient features is applied to build a line candidate pool, which probably contains many false detection results. Then, hybrid feature filtering is designed to pick the horizon line segments from the pool in the fine step. Finally, the fine line segments are stitched to obtain the whole horizon line based on random sample consensus (RANSAC). Using real data in the maritime environment, the experimental results demonstrate the effectiveness of CFS, compared to the existing methods in terms of accuracy and robustness.

## 1. Introduction

Currently, with the rapid development of artificial intelligence technology, unmanned surface vehicles (USVs) have become more and more important in maritime systems [1,2,3]. Compared with manned surface vehicles, USVs are more easily manipulated and are more adaptable to the environment with lower risk to staff. USVs have been applied in various military and civilian missions, such as science, mapping, defense and general robotics research [4,5,6]. The vision sensors equipped on the USVs usually are used to perceive surrounding information, which plays an important role in guaranteeing the safety and efficiency of the USVs without human intervention.

The horizon line is an important information source for USVs. When considering real applications, the maritime horizon line can be utilized for autonomous navigation, attitude estimation, obstacle detection, and target tracking, for example [7,8]. However, maritime horizon line detection is quite challenging due to the following two points. First, the pixel points of the horizon line features are far fewer than the pixel points of the entire image; and second, horizon line detection suffers from the complex maritime environment, waves, light changing and partial occlusions by maritime vessels or islands, for example. Sometimes, the horizon line in maritime application is blurry since the image of the sky and the sea is similar.

During the last few decades, horizon line detection has been attracting more attention in the community of computer vision and USV applications. The existing research can be divided into methods using local features and methods using global features.

The local features methods usually regard the horizon line as a line segment using local features, such as edge detection. Kim et al. proposed a horizon line detection method with the combination of Canny edge detection and the Hough transform [9]. However, the Hough transform is time-consuming, which might not meet the real-time requirement in maritime applications. Tang et al. used the Radon transform for horizon detection for infrared ship detection applications [10], but the result might be influenced by false line segments as the Radon transform cannot determine the endpoints of line segments. A detailed comparison of methods using local features for horizon line detection in maritime images is shown in Libe’s work [11]. Prasad et al. presented a horizon line detection method named MuSCoWERT [12]. First, their method detects the long linear features consistent over multiple scales using multi-scale median filtering of the image. Then, the Radon transform on a weighted edge map is applied to detect linear features. Prasad et al. presented another multi-scale approach, MSCM-LiFe [13]. Using that method, the Hough transform, and intensity gradients are used to find the line feature candidates. Although these methods using local features are effective for line segment extraction, the major shortcoming is that they are unable to distinguish the horizon line from the similar line segments in the sea image.

The methods in the other category use global features to calculate an optimization criterion for different candidate line positions and orientations using the features of the whole image. Wang et al. proposed a sea-sky line detection method based on global gradient saliency [14], but it still suffers from false detection when some straight lines are in the image, such as waves. Gershikov analyzed the performance of horizon line detection using color features and showed that color information can be justified when smaller angular and height deviations are of primary importance [15]. However, there is little discussion of the problem of partial occlusion. Segmentation methods are also used to obtain the horizon line by segmenting the image into the sea/water and the sky/ground. Scherer et al. proposed a water detection method from a flying robot [16] where the onboard inertial measurement unit (IMU) is used to obtain the horizon line in order to obtain a color distribution of the regions below and above the horizon. The image is then segmented to extract the water region using the classifier. Fefilatyev et al. proposed a horizon line detector for the recognition and tracking of marine vehicles in videos [17]. Prior to trying to find the horizon line, their method generates a preliminary segmentation of objects by creating binary maps where the location of a possible target object is defined by non-zero elements. A statistical horizon line detection algorithm is then proposed, which attempts to minimize the intra-class variance of the sea/ground and sky distributions. However, the fundamental drawback of that method is that it approximates the edge of water by a horizon line and cannot handle situations in coastal waters [18]. Kristan et al. addressed the problem of online detection by constrained unsupervised segmentation [18]. To achieve this, a semantic segmentation model (SSM) is proposed for structurally constrained semantic segmentation with application to USV obstacle-map estimation.

More recently, a deep neural network has been applied for horizon line detection. Jeong et al. proposed a horizon detection method for maritime scenes using a scene parsing network [19]. They also proposed a horizon detection method that combines a multi-scale approach and a convolutional neural network [20]. However, the generalization of these methods still needs to be further investigated.

The aim of this paper is to propose a robust maritime horizon line detection method, coarse-fine-stitched (CFS), for USV applications. CFS is mainly composed of three steps. The first step of CFS is the coarse detection, in which a line segment detection approach in which gradient features are applied to extract all the line segments in the image to build a line candidate pool. Although there are a few missing detections in the candidate pool, it probably contains many false detection results due to the background environment. To solve this problem, using the fine step of CFS, hybrid feature filtering is designed to select the horizon line segments from the pool. To improve the performance of the fine step, the distinguished features of the horizon line segments are modeled to build hybrid feature filtering, including color and morphology. Finally, a line segment stitching method based on random sample consensus (RANSAC) is proposed to obtain the whole horizon line. Considering the linear relationship of the horizon line segments, the RANSAC method is used to stitch the true results and remove the negative line segments, which can improve the robustness of this approach in a complex maritime environment.

The remainder of this paper is organized as follows. The details of the proposed CFS method are presented in Section 2. Section 3 discusses the experiments used to demonstrate the practical utility of this approach. Finally, the conclusions are shown in Section 4.

## 2. Coarse-Fine-Stitched Method

### 2.1. Framework

**Figure 1:**
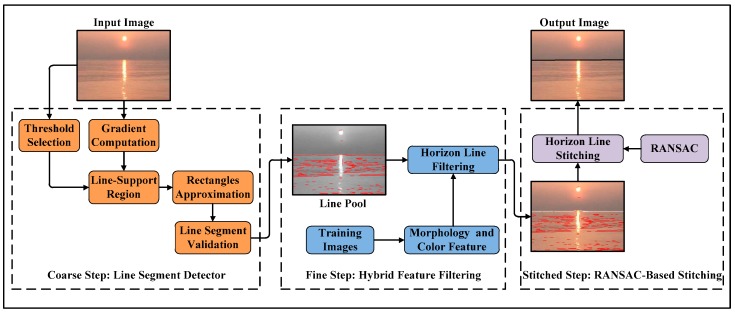
The framework of the coarse-fine-stitched (CFS) method for unmanned surface vehicle (USV) applications. The first step of the method is a problem-specific design line segment detector, the second step is a horizon line filter, and the third step is a random sample consensus-based (RANSAC-based) line segment stitching method.

The framework of the proposed coarse-fine-stitched (CFS) method for unmanned surface vehicle (USV) applications is presented in Figure 1. It operates in a three-step way. During the coarse step, the image gradient features are exploited to obtain all the lines of the image, which are all candidates for horizon line segments. In this step, a problem-specific design line segment detection (LSD) algorithm is proposed to extract all the candidates with a short processing time, generating a line pool. Although the coarse detection method can obtain the horizon line candidates of the image, some non-horizon line segments could be selected into the pool. Therefore, in the fine second step of this method, a horizon line filter is designed to eliminate the non-horizon lines from the pool by using hybrid feature filtering, which was designed based on the morphological and color features of the horizon line. Finally, to achieve the accurate identification of the horizon line, the horizon line segments are stitched in this step. Specifically, a random sample consensus-based (RANSAC-based) line segment stitching method is proposed to obtain the whole horizon line, which can improve the robustness of this approach in a complex maritime environment.

### 2.2. Coarse Detection

Line features are one of the most important features that determine the effectiveness of the coarse-fine-stitched (CFS) method. von Gioi et al. presented a line segment detection (LSD) algorithm which gave accurate results with fast computational speed [21]. The LSD algorithm extracts line segments from an image in three steps: (1) Line-support region: Group connected pixels with gradient values exceeding a threshold into line-support regions by applying a region-growing algorithm; (2) Rectangle approximation: Find the rectangle that best approximates each line-support region; (3) Line segment validation: Validate each rectangle based on a contrary method, which counts the number of aligned points (points with a gradient direction approximately orthogonal to the line segment) and finds the line segments as outliers.

However, the direct application of the LSD algorithm is not appropriate for the horizon line detection problem for the following two reasons.

**Figure 2:**
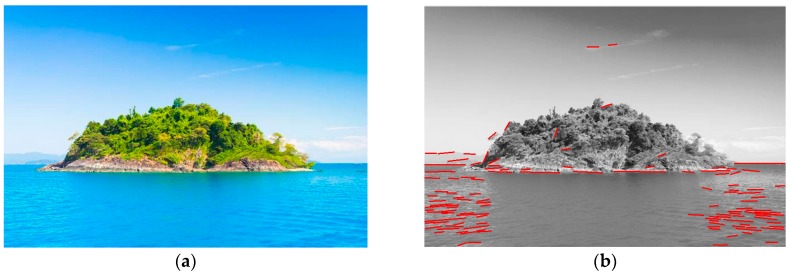
The direct application of the line segment detection (LSD) algorithm with false positive: (**a**) A given image with a small island, clouds and waves; (**b**) Line segments of the island, the clouds and the waves in the image also are extracted by the LSD algorithm, causing a false positive.

**Figure 3:**
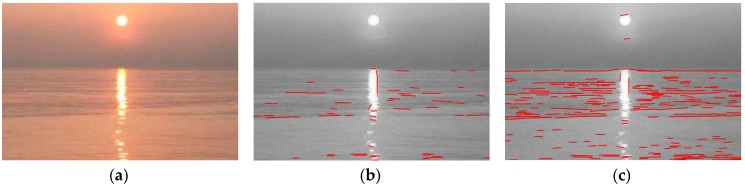
The direct application of the LSD algorithm with a false negative: (**a**) A given image with a horizon line that is very background-like due to vapor and illumination; (**b**)The horizon line is ignored totally by the LSD algorithm, causing a false negative; (**c**) The results of this problem-specific design LSD for coarse detection.

(1) LSD cannot distinguish the horizon line from other line segments because it just focuses on detecting line segments [22,23,24]. A line that is not the horizon line also will be obtained. Figure 2b gives the result of using LSD for a given image, lines of the island, the clouds and the waves in the image are also extracted, causing a false positive. Therefore, to accurately extract the horizon line from a given image, a filter method should be considered to eliminate non-horizon line segments from all line segments.

(2) LSD ignores the horizon line that is background-like, which might cause a significant false negative. Figure 3b shows the horizon line is totally ignored. During the first step of the LSD algorithm, to improve the exactness of line segment detection, a gradient threshold is selected to eliminate pixels with a small gradient magnitude because the orientation error cannot be ignored for a small gradient magnitude [25], which can create a linear pattern and thereby cause false detections. The threshold on the gradient magnitude in the LSD algorithm is set as [21]:

(1) $$T_{g}^{\prime }=\frac{2}{\sin22.5^{{}^{\circ}}}=5.3$$

Pixels with a gradient magnitude smaller than the threshold will not be regarded as part of any line segment. However, in this horizon line detection task, the horizon line obtained by the USV might be very background-like, due to vapor or illumination, for example. Their corresponding pixels with gradient magnitude might be under the threshold often. This leads to the horizon line being ignored and causes high false negatives. Figure 3b shows the horizon line is ignored totally in this step, since the gradient magnitude of the pixels do not exceed $T_{g}^{\prime }$.

Considering that false positive can be processed further in the next step, a smaller threshold is set to avoid ignoring pixels with background-like horizon lines in this step. Demonstrated in the analysis above and inspired by the LSD algorithm, the LSD framework is applied and then the current method focuses on how to redesign the gradient threshold in the first step of the algorithm. A problem-specific LSD design then is proposed to obtain all the lines of a given image.

Regarding the gradient threshold selection problem, an intensity-based method is applied to set the threshold [26]:

(2) $$T_{g}=\frac{\mu_{mean}}{\mu_{\max}}$$

where $\mu_{mean}$ is the mean gradient value of the image, and $\mu_{\max}$ is the maximum of the mean gradient value of the grid images. The authors divided the image into an 8 × 8 grid. It represents an adaptive threshold that depends on the gradient value of the image. It is evident that $T_{g}\leq 1\leq T_{g}^{\prime }$, thus, with the smaller threshold, pixels of the background-like horizon line segments will not be ignored in the first step of this method, as shown in Figure 3c.

The problem-specific LSD design is able to extract all the lines of a given image and generate a line pool, ***C****_h_*, which is composed of all candidates for horizon line segments, denoted as ***l***_1_, ***l***_2_, …, ***l****_Nc_*, where *N_c_* is the total number of line segments obtained by the coarse detection method, resulting in $\left\{l_{i}|l_{i}\in C_{h},\;i\in [1,N_{c}]\right\}$.

### 2.3. Fine Detection

Subsequent to the process of the first step, a line pool ***C****_h_* is obtained. However, some elements of ***C****_h_* might be background noise causing a sufficient false positive. To avoid the interference of the non-horizon line, a horizon line filter is applied to suppress the noise and extract horizon line segments from the line pool ***C****_h_*. The non-horizon line is filtered out from the pool according to the distinguished features of the horizon line segments, including color and morphology.

#### 2.3.1. Morphology Feature

The morphology feature of the horizon line includes the length and direction properties, as well as others [7]. However, in some environments, the horizon line detection might be obscured partially by maritime vessels or islands, as shown in Figure 2a. Thus, the length property would not perform well for horizon line detection. A new direction property is used for fine detection in this paper.

Denote the equation of the line horizon segment ***l****_i_* as:

(3) $$l_{i}:y-k_{i}x-b_{i}=0$$

where *k_i_* and *b_i_* are the gradient and the intercept of the line horizon segment ***l****_i_*, respectively.

Denoting the direction of the horizon line segment ***l****_i_* as *d_i_*:

(4) $$d_{i}=\tan^{-1}k_{i}$$

Since the camera system is usually fixed on the unmanned surface vehicle (USV), the direction of the horizon line obtained by the camera system is approximately equal to the roll angle of the USV *β*. When used in real applications, the angle error between the roll angle of the USV and the direction of the horizon line is the alignment error, which can be measured earlier, denoted as *e*. Subsequently, the result is:

(5) $$\left|d_{i}-\beta -e\right|\leq \delta$$

where δ is a very small angle value, which is caused by other factors, such as structural deformation.

Denote the maximum value of the roll angle as *β*_max_:

(6) $$-\delta -\beta_{\max}+e\leq d_{i}\leq \delta +\beta_{\max}+e$$

*β*_max_ is set as 30°, and the USV does not risk capsizing.

#### 2.3.2. Color Feature

**Figure 4:**
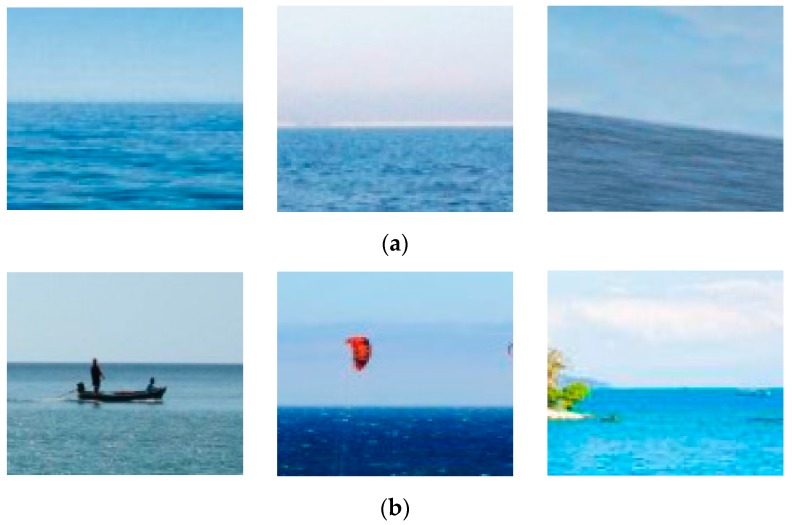

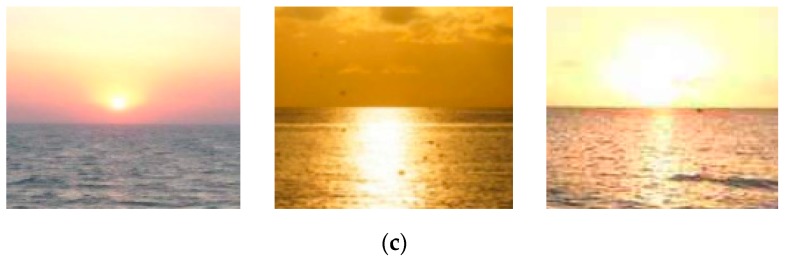
The color feature of the horizon line segments: (**a**) The image examples contain the horizon line with blue sea and white/blue sky; (**b**) The image examples contain the horizon line with blue sea, white/blue sky and other objects, such as boat, bird, and island; (**c**) The image examples contain the horizon line with the setting sun scene.

The color feature is another essential feature to distinguish the horizon line segments from other line segments. Since the horizon line is the apparent edge that separates sea from sky, the greater the color feature difference of the sea and the sky, the easier it is to distinguish the horizon line segments and other line segments. Figure 4 shows the image examples containing the color feature of the horizon line segments. The color feature between the two sides of the horizon line segments is distinguished.

First, there is a notable color difference between the two sides of the horizon line segments. The greater the color feature variance of the two sides of a horizon line segment, the easier it is to distinguish the horizon line segments and other line segments. Second, the color of each side of the horizon line segment is composed of several categories, for example, the color of the up-side of a horizon line segment is probably blue, white, gray, red, yellow, and more. The color of the down-side of the horizon line segment is probably blue, gray, etc. Third, on the same side of the horizon line segment, the color and texture are roughly the same and, the closer to the horizon line segment, the more important it is to distinguish the result.

**Figure 5:**
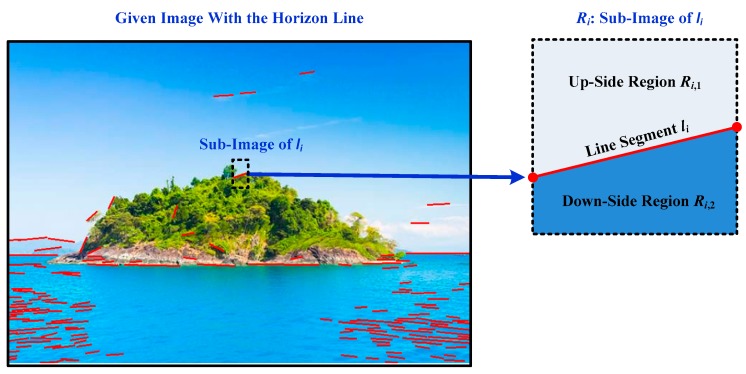
Given an image with a horizon line, the line segment pool is obtained through the coarse detection step. The sub-image of line segment then is extracted for fine detection, whose color feature is calculated to identify whether it is the horizon line segment.

Given an image with a horizon line, the line segment pool is obtained through the coarse detection step. The sub-image of line segment ***l****_i_* is extracted for fine detection, denoted as ***R****_i_*. The sub-image with a line segment detected by the coarse detection step is shown in Figure 5. The up-side region and down-side region of line segment ***l****_i_* is denoted as ***R****_i,_*_1_ and ***R****_i,_*_2_, respectively, with the sub-image of ***l****_i_*, the color feature is calculated to identify whether it is the horizon line segment.

Concerning the current method, the first three color moments of the sub-image of ***l****_i_* are used for fine detection, which characterize the color distribution of sub-images. It is scaling and rotation invariant. Moreover, since color moments encode both shape and color information, they are a good feature to use under changing lighting conditions [27]. The first three color moments of sub-image ***R****_i_* are obtained as follows:

##### Mean

The first color moment of region ***R****_i,_*_1_ and region ***R****_i,_*_2_ can be calculated as the average color in the image region:

(7) $$\mu_{k,1}^{i}=\frac{1}{N_{i,1}}\sum_{j=1}^{N_{i,1}}p_{k,1,j}^{i}$$

(8) $$\mu_{k,2}^{i}=\frac{1}{N_{i,2}}\sum_{j=1}^{N_{i,2}}p_{k,2,j}^{i}$$

where *N_i,_*_1_ and *N_i,_*_2_ are the number of pixels of region ***R****_i,_*_1_ and region ***R****_i,_*_2_, respectively. $p_{k,1,j}^{i}$ and $p_{k,2,j}^{i}$ are the value of the *j*th pixel of region ***R****_i,_*_1_ and region ***R****_i,_*_2_ at the *k*th color channel, respectively. The color image is processed and *k =* 1, 2, 3, corresponding to the three color channels of the image.

##### Standard Deviation

The second color moment is the standard deviation. The second color moment of region ***R****_i,_*_1_ and region ***R****_i,_*_2_ can be obtained by taking the square root of the variance of the color distribution:

(9) $$\sigma_{k,1}^{i}=\sqrt{\frac{1}{N_{i,1}}\sum_{j=1}^{N_{i,1}}{\left(p_{k,1,j}^{i}-\mu_{k,1}^{i}\right)}^{2}}$$

(10) $$\sigma_{k,2}^{i}=\sqrt{\frac{1}{N_{i,2}}\sum_{j=1}^{N_{i,2}}{\left(p_{k,2,j}^{i}-\mu_{k,2}^{i}\right)}^{2}}$$

##### Skewness

The third color moment is the skewness. It measures how asymmetric the color distribution is and, thus, it gives information about the shape of the color distribution. Skewness of region ***R****_i,_*_1_ and region ***R****_i,_*_2_ can be computed with the following formula:

(11) $$s_{k,1}^{i}=\sqrt[{3}]{\frac{1}{N_{i,1}}\sum_{j=1}^{N_{i,1}}{\left(p_{k,1,j}^{i}-\mu_{k,1}^{i}\right)}^{3}}$$

(12) $$s_{k,2}^{i}=\sqrt[{3}]{\frac{1}{N_{i,2}}\sum_{j=1}^{N_{i,2}}{\left(p_{k,2,j}^{i}-\mu_{k,2}^{i}\right)}^{3}}$$

Using Equation (7) to Equation (12), the color feature vector of region ***R****_i,_*_1_ and region ***R****_i,_*_2_ can be obtained as the nine-dimension vectors:

(13) $$c_{i,1}={\left[\mu_{1,1}^{i},\mu_{2,1}^{i},\mu_{3,1}^{i},\sigma_{1,1}^{i},\sigma_{2,1}^{i},\sigma_{3,1}^{i},s_{1,1}^{i},s_{2,1}^{i},s_{3,1}^{i}\right]}^{T}$$

(14) $$c_{i,2}={\left[\mu_{1,2}^{i},\mu_{2,2}^{i},\mu_{3,2}^{i},\sigma_{1,2}^{i},\sigma_{2,2}^{i},\sigma_{3,2}^{i},s_{1,2}^{i},s_{2,2}^{i},s_{3,2}^{i}\right]}^{T}$$

Using the color feature vectors, the color feature difference between region ***R****_i,_*_1_ and region ***R****_i,_*_2_ can be calculated as:

(15) $$d(c_{i,1},c_{i,2})=\sum_{k=1}^{3}\left(w_{\mu }\left|\mu_{k,1}^{i}-\mu_{k,2}^{i}\right|+w_{\sigma }\left|\sigma_{k,1}^{i}-\sigma_{k,2}^{i}\right|+w_{s}\left|s_{k,1}^{i}-s_{k,2}^{i}\right|\right)$$

where $w_{\mu }$, $w_{\sigma }$ and $w_{s}$ are the weights for each of the three color moments used.

The color feature difference between region ***R****_i,_*_1_ and region ***R****_i,_*_2_ will be used to compute a difference score. Considering that the horizon line is the apparent edge that separates sea from sky, the value of $d(c_{i,1},c_{i,2})$ should be larger than threshold *T_d,_*_1_:

(16) $$d(c_{i,1},c_{i,2})\geq T_{d,1}$$

Since the color of each side of the horizon line segment is composed of several categories, the color feature difference between region ***R****_i,_*_1_ (or region ***R****_i,_*_2_) and the training data is calculated, which should be smaller than threshold *T_d,_*_2_:

(17) $$d(c_{i,m},c_{i,m,t})\leq T_{d,2}$$

where *m* = 1, 2, corresponding to the up-side region ***R****_i,_*_1_ and down-side region ***R****_i,_*_2_, respectively. 

The training data is composed of 1000 images including the horizon line with different illumination, rain, fog, and occlusion, for example. The authors have *T_d_*_,1_ = 1.5, and *T_d_*_,2_ = 1 used in these experiments.

**Figure 6:**
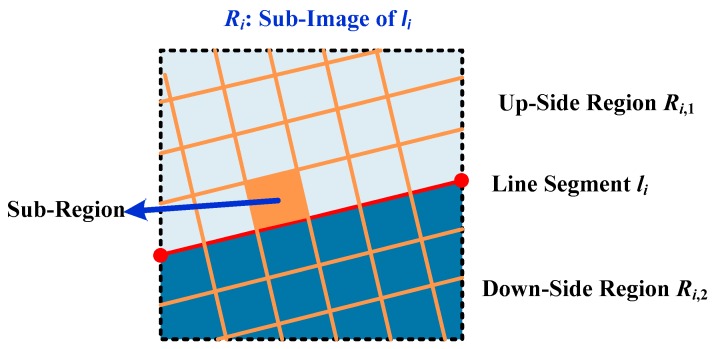
The up-side region ***R**_i,_*_1_ and down-side region ***R****_i,_*_2_ are divided into a series of sub-regions.

To improve the effectiveness of the coarse-fine-stitched (CFS) method, the up-side region ***R****_i,_*_1_ and down-side region ***R****_i,_*_2_ are divided into a series of sub-regions, as shown in Figure 6.

Similar to the color moments of region ***R**_i,_*_1_ and region ***R**_i,_*_2_, the color feature vector of each sub-region can be calculated as:

(18) $$c_{i,1,p}={\left[\mu_{1,1,p}^{i},\mu_{2,1,p}^{i},\mu_{3,1,p}^{i},\sigma_{1,1,p}^{i},\sigma_{2,1,p}^{i},\sigma_{3,1,p}^{i},s_{1,1,p}^{i},s_{2,1,p}^{i},s_{3,1,p}^{i}\right]}^{T}$$

(19) $$c_{i,2,p}={\left[\mu_{1,2,p}^{i},\mu_{2,2,p}^{i},\mu_{3,2,p}^{i},\sigma_{1,2,p}^{i},\sigma_{2,2,p}^{i},\sigma_{3,2,p}^{i},s_{1,2,p}^{i},s_{2,2,p}^{i},s_{3,2,p}^{i}\right]}^{T}$$

where $c_{i,1,p}$ and $c_{i,2,p}$ are *p*th sub-regions of region ***R****_i,_*_1_ and region ***R****_i,_*_2_, respectively.

Denote the color feature difference of two sub-regions on the same side as $d(c_{i,m,p},c_{i,m,q})$. As on the same side of the horizon line segment the colors are roughly the same, then the color feature differences of the two sub-regions on the same side should satisfy:

(20) $$\mathrm{var}\left(d(c_{i,m,p},c_{i,m,q})\right)\leq T_{d,3}$$

where $\mathrm{var}\left(d(c_{i,m,p},c_{i,m,q})\right)$ is the variance of $d(c_{i,m,p},c_{i,m,q})$ on the same side of the horizon line segment. The threshold *T_d,_*_3_ is set as 1.

Corresponding to the discussion in this section, hybrid feature filtering is designed and the line segments of the line pool that satisfy Equation (6), Equation (16), Equation (17) and Equation (20) are picked as the horizon line segments for the next step of CFS.

### 2.4. Robust Stitching

Through the fine detection step of the coarse-fine-stitched (CFS) method, line segments are picked from the line pool as the horizon line segments, denoted as ${\tilde{l}}_{1},{\tilde{l}}_{2},\ldots ,{\tilde{l}}_{N_{f}}$, where *N_f_* is the total number of line segments obtained by the fine detection method. Since these candidate horizon line segments have already passed the hybrid feature filtering, most of them have the typical characteristics of the horizon line. However, there are still two problems that need to be resolved to obtain the horizon line result for unmanned surface vehicle (USV) applications.

First, some parts of the horizon line might be missing due to the occlusion of islands and vessels, illumination, or fog, for example. Using this scenario, the horizon line is divided into several pieces of horizon line segments, rather than a complete line.

Second, although most of the line segments are the horizon line segments through fine detection, there inevitably might be some non-horizon line segments, which will have a negative impact on the final result.

To address these challenges, a random sample consensus-based (RANSAC-based) line segment stitching method is proposed to obtain the whole horizon line. Found in real applications, the horizon line is a straight line. Even if it is divided into several pieces, there is still a linear relationship between them. Therefore, a natural idea is that the horizon line can be obtained through straight line fitting, which is used to estimate the best line passing through the candidate line segments after fine line detection. Considering the fine step, it is difficult to completely exclude a false positive, therefore a RANSAC algorithm is applied in this paper for robust line fitting. The advantage of RANSAC is the ability to use redundant consistency to fit the correct results and to reduce the effect of a false positive on the final results [28].

Denote the equation of the horizon line ***L*** as:

(21) L:y−kx−b=0

where *k* and *b* are the gradient and the intercept of the line horizon ***L***, respectively the parameters *k* and *b* are to be determined as follows.

Denote the equation of the line segment ${\tilde{l}}_{i}$ as:

(22) $${\tilde{l}}_{i}:y-{\tilde{k}}_{i}x-{\tilde{b}}_{i}=0$$

where $i\in [1,N_{f}]$, ${\tilde{k}}_{i}$ and ${\tilde{b}}_{i}$ are the gradient and the intercept of the line horizon segment ${\tilde{l}}_{i}$, respectively. The two end points of the line segment ${\tilde{l}}_{i}$ are ${\left[x_{i1},y_{i1}\right]}^{T}$ and ${\left[x_{i2},y_{i2}\right]}^{T}$ in image coordinates, and the position of its midpoint is ${\left[{\bar{x}}_{i},{\bar{y}}_{i}\right]}^{T}={\left[\left(x_{i1}+x_{i2}\right)/2,\left(y_{i1}+y_{i2}\right)/2\right]}^{T}$.

The stitching method is used to solve the two parameters in Equation (21) by minimizing the total mean distance between the line segment ${\tilde{l}}_{1},{\tilde{l}}_{2},\ldots ,{\tilde{l}}_{N_{f}}$ and the horizon line results:

(23) $$\underset{k,b}{\min}\left\{\sum_{i=1}^{N_{f}}\left\|l_{i}\right\|d_{i}\right\}$$

where *d_i_* is the distance between the midpoint ${\left[{\bar{x}}_{i},{\bar{y}}_{i}\right]}^{T}$ of line segment ${\tilde{l}}_{i}$ and the horizon line results:

(24) $$d_{i}=\frac{{\bar{y}}_{i}-k{\bar{x}}_{i}-b}{\sqrt{k^{2}+1}}$$

and $\left\|l_{i}\right\|=\sqrt{{\left(x_{i1}^{}-x_{i2}^{}\right)}^{2}+{\left(y_{i1}^{}-y_{i2}^{}\right)}^{2}}$ is the weight of the distance, which means the result is intended to choose the long-line segment candidates as the final results. 

This illustrated that the optimal function is a continuous convex function, which can be solved easily by the gradient descent method [29]. Using all line segment candidates, the horizon line detection result can be solved, denoted as ***L***_0_. 

Although most of the line segments are horizon line segments through fine detection, there inevitably might be some non-horizon line segments, which will have a negative impact on the final result. To solve this problem, RANSAC is applied for robust stitching. Batches of line segment candidates are randomly selected to solve the equation of the horizon line, denoted as ***L****_j_*, which is the *j*th iteration of RANSAC.

**Figure 7:**
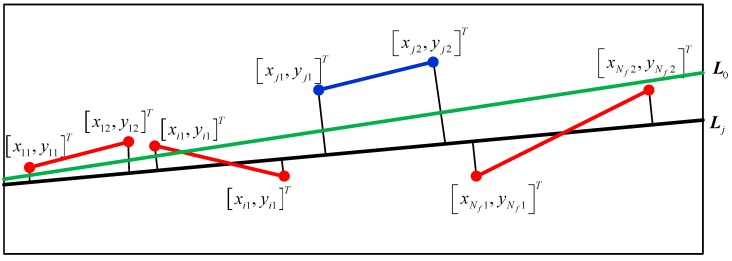
RANSAC-based line segment stitching method. ***L***_0_ is the line fitting results using all line segment candidates and ***L****_j_* is the RANSAC-based line fitting.

The principle of the RANSAC-based line segment stitching method is shown in Figure 7. ***L***_0_ is the line fitting result using all line segment candidates and ***L****_j_* is the RANSAC-based line fitting. The distance between the midpoint of the line segment candidate ${\tilde{l}}_{j}$ and ***L****_j_* is larger than the threshold, which is marked as an outlier and excluded for stitching.

Looking at the batch, each distance between the midpoint of the line segment candidate and ***L****_j_* is calculated. When the distance is smaller than a threshold, the line segment is marked as an in-line candidate; otherwise, the line segment is marked as an outlier candidate. The batches of line segment candidates are randomly selected to obtain a batch with the largest number of inline candidates.

The main procedures of the CFS method are summarized as follows:

Step 1. Coarse detection
  1.1 Calculate gradient threshold *T_g_* using Equation (2)
  1.2 Implement LSD algorithm with *T_g_* and finding the line pool ***C****_h_*
Step 2. Fine detection
  2.1 Calculate the direction of the line segments in ***C****_h_* using Equation (4)
  2.2 Calculate the color feature vector of the sub-images using Equations (13)–(14)
  2.3 Calculate the color feature vector of the sub-regions using Equations (18)–(19)
  2.4 Select the line segments of ***C****_h_* that satisfy Equations (6), (16), (17) and (20)
Step 3. Robust stitching
  3.1 Execute RANSAC and solve Equation (23)

## 3. Results and Discussion

Three separate experiments were designed to evaluate the practical utility of the coarse-fine-stitched (CSF) method. The first experiment was to evaluate the performance of the CFS method for images with different backgrounds. The second experiment was the parameter sensitivity analysis. The third experiment was to test the performance of the method compared with existing methods in terms of accuracy and time-consumption.

The experiments were conducted using a PC with Intel Core i3-6006U CPU 2.00 GHz and 4 GB memory. The testing data was composed of 500 testing images, the public dataset Singapore Maritime Dataset (SMD) [13], and the public dataset Marine Obstacle Detection Dataset (MODD) [18]. The 500 testing images were acquired using a digital camera (Sony-DSC-T70) in Weihai City, China, in July, 2017. The resolution was 640 × 800 pixels, with different backgrounds. Half of the images contained mist or fog and the other half contained relatively clear horizon lines. Additionally, to test the performance of this method, the public dataset SMD and MODD were used for comparison. The training images were composed of more than 1000 images with the horizon line downloaded from http://image.baidu.com/, with different illumination, rain, fog, and occlusion, etc.

### 3.1. Coarse-Fine-Stitched Detection Results with Different Backgrounds

High visual complexity made it difficult to detect the horizon line that was not salient. The authors divided the testing image based on its clutter measure into *K* sub-images [30]. The measure was based on the mean and standard deviation (μ,σ) of the intensity distribution of the pixels:

(25) $$\mu_{n}=\frac{1}{N}\sum_{(x,y)\in S_{n}}I(x,y)$$

where I was a given image, (x,y) indicated the pixel index. N was the total number of pixels in sub-image $S_{n}$.

(26) $$\sigma_{n}=\sqrt{\frac{1}{N-1}\sum_{(x,y)\in S_{n}}{\left(I(x,y)-\mu_{n}\right)}^{2}}$$

The clutter measure of an image was given by the average of the intensity variance of the sub-images:

(27) $$clutter=\sqrt{\frac{1}{K}\sum_{n=1}^{K}\sigma_{n}^{2}}$$

The authors divided the image into 4 × 4 sub-images with reference to Candamo’s work [31]. The mean clutter of the database was denoted as μ. When the clutter of an image was less than μ, it was considered as low clutter. To test the validation of this approach, the authors used the public datasets Singapore Maritime Dataset (SMD) and Marine Obstacle Detection Dataset (MODD) for benchmarking. The clutter of these images (or frames in video) varied from 1 to 45, and μ=14. The processing time for an image was a fraction of a second, which permitted the authors to test the method on thousands of images.

**Figure 8:**
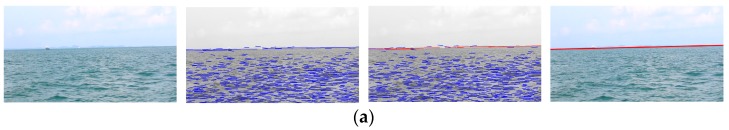

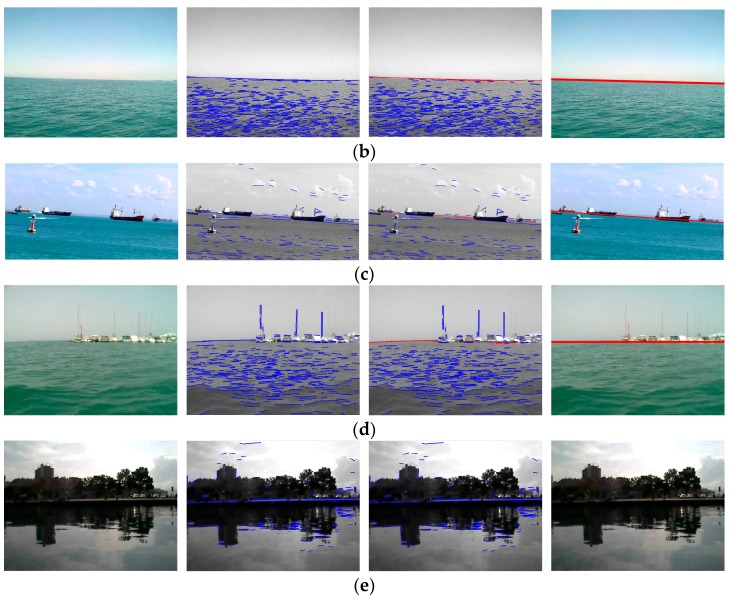
CFS detection results for the images from Singapore Maritime Dataset (SMD) and Marine Obstacle Detection Dataset (MODD) with different backgrounds. The first column is the original images and the next three columns are the output of the coarse detection, fine detection, and robust stitching of CFS, respectively: (**a**) Test results for a low clutter image (*clutter =* 11) with the horizon line from SMD; (**b**) Test results for a low clutter image (*clutter =* 11) with the horizon line from MODD; (**c**) Test results for a high clutter image (*clutter =* 17) with the horizon line from SMD; (**d**) Test results for a high clutter image (*clutter =* 17) with the horizon line from MODD; (**e**) Test results for a high clutter image (*clutter =* 41) without the horizon line from MODD.

Coarse-fine-stitched (CFS) detection results with a low clutter image (*clutter* < μ) and high clutter image (*clutter* ≥μ) are shown in Figure 8. The intermediate process of the CFS method is shown to illustrate their effects. The second, third, and forth column were the output of the coarse detection, fine detection, and robust stitching of CFS, respectively. Figure 8a–d show that the experimental results demonstrated that the method was able to extract the horizon line accurately with different backgrounds. The experiment also showed that there were few false positives for images without the horizon line, even though the background noise was large (Figure 8e).

### 3.2. Parameter Sensitivity Analysis

**Figure 9:**
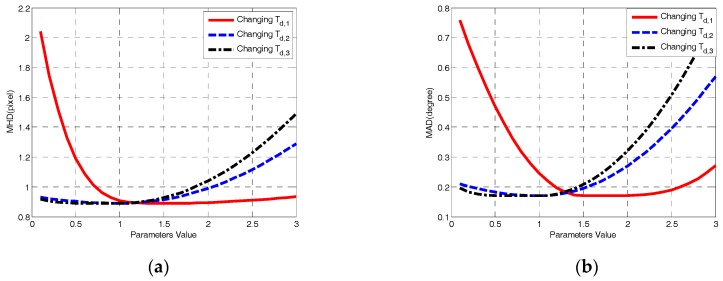
Parameter sensitivity analysis: (**a**) Mean height deviation (MHD) with changing control parameters. (**b**) Mean angle deviation (MAD) with changing control parameters.

There were three main control parameters in the fine detection of the coarse-fine-stitched (CFS) method, such as threshold *T_d,_*_1_ = 1.5, threshold *T_d,_*_2_ = 1 and threshold *T_d,_*_3_ = 1. By fixing two parameters and changing another parameter, the mean height deviation (MHD, pixels) and mean angle deviation (MAD, degrees) of CFS performed on the testing data are shown in Figure 9. Setting a smaller *T_d,_*_1_ resulted in a larger probability of selecting the line segments, even if the difference between the regions was not distinct enough. Setting a larger *T_d,_*_2_ or *T_d,_*_3_ resulted in a larger probability of picking the line segments, even if the similarity between the sub-regions was not enough. Both of these caused false positives and decreased the detection accuracy.

### 3.3. Methods Comparision

To test the performance of this method compared with the state-of-the-art methods, horizon line detection methods with a processing time for an image that was less than one second was applied. To verify this method, the authors used Wang’s algorithm [14], MSCM-LiFe [13], and SSM [18] as the baseline comparisons for the work. The dataset, the Singapore Maritime Dataset (SMD), and Marine Obstacle Detection Dataset (MODD), were applied for comparison in this section.

**Figure 10:**
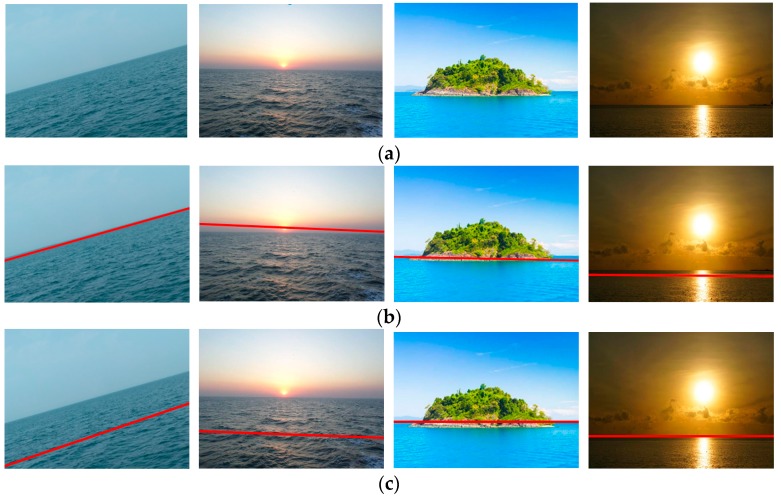

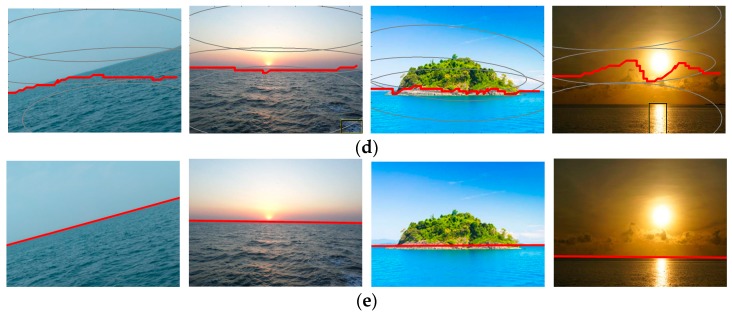
A comparison of various horizon line detection methods using the authors’ testing data: (**a**) Original image; (**b**) Wang’s algorithm; (**c**) MSCM-LiFe; (**d**) SSM; (**e**) CFS method.

The performance of the baseline methods and this approach using the authors’ dataset in a maritime environment is shown in Figure 10. The figure illustrates that the proposed coarse-fine-stitched (CFS) method can detect the horizon line more accurately and robustly in the presence of various interfering factors, when compared with the other baseline methods. When using the testing image with a relatively simpler background, such as the first column in Figure 10, most of the methods obtained the horizon line accurately. However, when there were several interfering factors, such as illumination changing, clouds, or islands, the CFS method performs best when compared with the other methods.

**Figure 11:**
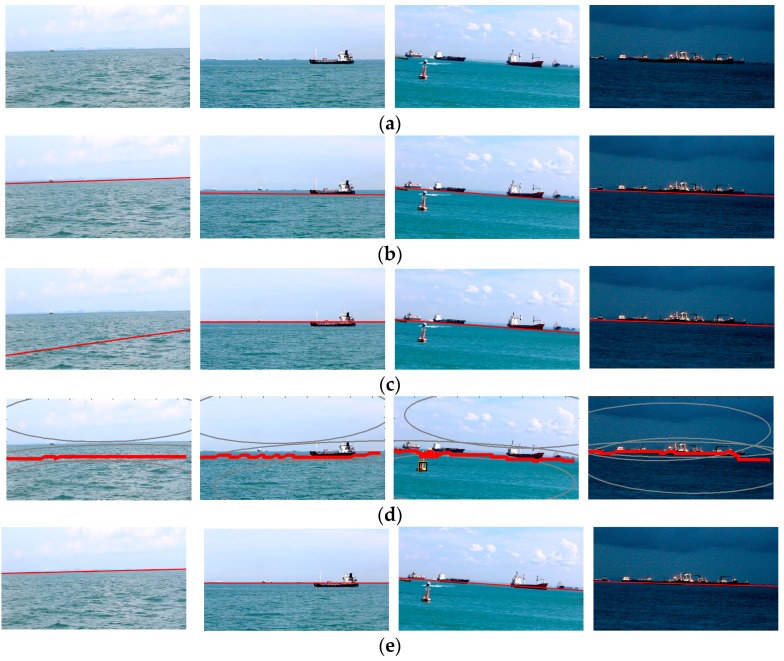
A comparison of various horizon line detection methods using SMD: (**a**) Original image; (**b**) Wang’s algorithm; (**c**) MSCM-LiFe; (**d**) SSM; (**e**) CFS method.

The performance of the baseline methods and this approach using SMD is shown in Figure 11. Wang’s algorithm was affected easily by the bottom of the boats. SSM was very sensitive to obstacles when using the segmentation method. MSCM-LiFe was affected easily by waves, as shown in the first column of Figure 10c and Figure 11c. Compared with the other methods, the CFS method performed accurately and robustly for the testing data.

**Figure 12:**
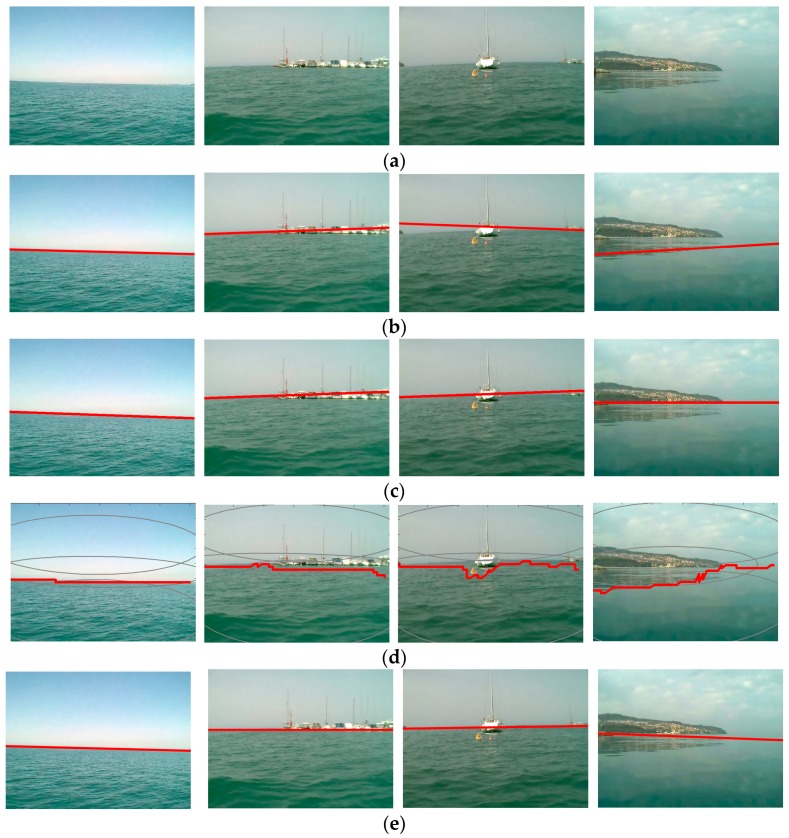
A comparison of various horizon line detection methods using MODD: (**a**) Original image; (**b**) Wang’s algorithm; (**c**) MSCM-LiFe; (**d**) SSM; (**e**) CFS method.

The performance of the baseline methods and this approach using MODD is shown in Figure 12. When using the testing image with a relatively simpler background, such as the first to third columns in Figure 12, most of the methods obtained the horizon line accurately. Compared with other methods, Wang’s algorithm and SSM were more sensitive to obstacles or shadow. MSCM-LiFe and this CFS method performed accurately and robustly for the testing data.

**Figure 13:**
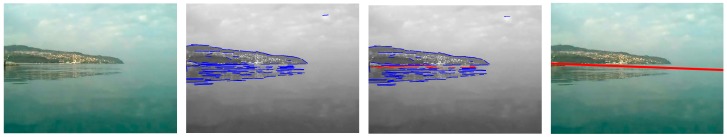
The performance of this CFS method on the very blurry horizon line. The first column is the original image, and the next three columns are the output of the coarse detection, fine detection, and robust stitching of CFS, respectively.

However, when the horizon line was very blurry, such as in the fourth column in Figure 12, this coarse detection method cannot detect the horizon feature and the result was affected entirely by the ground and its shadow. The intermediate process of the CFS method on the very blurry horizon line is shown Figure 13.

Table 1 shows the comparison of the horizon line detection performance on this test data. The detection errors consisted of mean height deviation (MHD) and mean angle deviation (MAD). The average computing time for each testing was counted for comparison. Table 1 shows that the MHD and MAD of this method was the smallest when compared with the three baseline methods. The MHD of this approach was 49.7%, 82.4% and 39.7% of the results of Wang’s algorithm, MSCM-LiFe, and SSM, respectively. The MAD of this approach was less than 0.2°, which was also the most accurate of all of the methods. Since SSM is the segmentation method, the results were not straight lines. Thus, the MAD of SSM was not calculated in Table 1. The experimental results show that the color feature and RANSAC of this approach played an important role in obtaining low false positives and accurate results. Due to the iteration in RANSAC, the average computing time for this method was 94 ms. The computing time of this method was a little longer than Wang’s algorithm and SSM, and was shorter than MSCM-LiFe. Since it included the time for the Hough transform and intensity calculation for the multi-scale median filtering of the image, the computing time of MSCM-LiFe was relatively longer than other methods.

## 4. Conclusions

To meet the requirement of horizon line detection for unmanned surface vehicle (USV) applications, a robust horizon line detection method, coarse-fine-stitched (CFS), was proposed in this paper. This CFS method operated in a three-step way. First, in the coarse step, a problem-specific design line segment detection (LSD) algorithm was presented to extract all the candidates, creating a line pool. Second, in the fine step, hybrid feature filtering was designed to select the horizon line segments from the pool. Finally, a random sample consensus-based (RANSAC-based) line segment stitching method was presented to obtain the whole horizon line. Experimental results demonstrate that the CFS outperformed the other state-of-the-art methods in terms of accuracy and robustness. Regarding the authors’ future work, a segmentation algorithm will be investigated further for horizon line and obstacle detection in USV applications.

## Figures and Tables

**Table 1:** Horizon line detection results using the current test data.

Algorithm	MHD	MAD	Average Computing Time
Wang’s algorithm	1.79	0.38°	57 ms
MSCM-LiFe	1.08	0.23°	231 ms
SSM	2.24	--	27 ms
CFS	0.89	0.19°	94 ms
